# MicroRNA-1224-5p Inhibits Metastasis and Epithelial-Mesenchymal Transition in Colorectal Cancer by Targeting SP1-Mediated NF-κB Signaling Pathways

**DOI:** 10.3389/fonc.2020.00294

**Published:** 2020-03-13

**Authors:** Jie Li, Wen Peng, Peng Yang, Ranran Chen, Qiou Gu, Wenwei Qian, Dongjian Ji, Qingyuan Wang, Zhiyuan Zhang, Junwei Tang, Yueming Sun

**Affiliations:** ^1^The First School of Clinical Medicine, Nanjing Medical University, Nanjing, China; ^2^Department of General Surgery, The First Affiliated Hospital of Nanjing Medical University, Nanjing, China

**Keywords:** colorectal cancer, metastasis, SP1, miR-1224-5p, NF-κB, prognostic biomarker

## Abstract

MicroRNAs (miRNAs) are small non-coding RNAs that play pivotal roles in cancer initiation and progression. However, the roles and molecular mechanisms of miRNAs in colorectal cancer (CRC) progression remain unclear. Here, we show that downregulation of miR-1224-5p in CRC is negatively correlated with SP1 expression and metastasis in patients and xenografted mouse models. Gain- and loss-of-function assays reveal that miR-1224-5p suppresses the migration, invasion, and epithelial–mesenchymal transition (EMT) of CRC cells *in vitro* and *in vivo* by directly targeting SP1. Moreover, SP1 promotes the phosphorylation of p65, which results in EMT progress in CRC cells. Clinical analysis reveals that miR-1224-5p and SP1 expression are remarkably associated with advanced clinical features and unfavorable prognosis of patients with CRC. Further study confirms that hypoxia accounts for the depletion of miR-1224-5p in CRC. The enhancement of hypoxia during epithelial–mesenchymal transition and metastasis of CRC cells is abolished by miR-1224-5p. Our findings provide the first evidence that miR-1224-5p is a potential therapeutic target and prognostic biomarker for patients with CRC.

## Introduction

Colorectal cancer (CRC), one of the most common malignant tumors of the digestive tract, is characterized by high incidence, high mortality, and poor prognosis. The incidence and mortality of CRC ranks third and second in cancer, respectively (1, 2). Despite advances in neoadjuvant therapy, radical surgery, post-operative chemoradiotherapy, and immunotherapy, the 5-year survival rate of patients with CRC remains disappointed because of distant metastases (3–7). Hence, revealing the molecular mechanism of CRC progression and metastasis and providing potential therapeutic targets of CRC are critical.

MicroRNAs (miRNAs) are small (~22 nt) non-coding RNAs that regulate gene expression through base pairing mostly with the 3′ untranslated regions (UTRs) of target messenger RNAs (mRNAs) (8). miRNAs have been reported to play crucial roles in tumor biological processes, including proliferation, apoptosis, migration, invasion, and differentiation (9). The association of non-coding RNAs especially miRNA dysregulation with the occurrence and development of cancer has been supported by evidence (10–13).

miR-1224-5p is found in human chromosome 3q27.1. The first study showing the relationship between miR-1224-5p and human cancer demonstrated that downregulated miR-1224 promotes cell proliferation in bladder cancer (14). Later, other studies revealed that miR-1224-5p is greatly decreased in intestinal-type gastric cancer and inhibits cell migration and metastasis by inhibiting focal adhesion kinase *in vitro* and *in vivo* (15). Nymark et al. reported that miR-1224-5p is remarkably decreased in asbestos-related lung cancer (16). In addition, miR-1224-5p acts as a tumor suppressor to repress cell proliferation and invasion and promote apoptosis by down-regulating the expression of CREB1 in malignant gliomas (17). Furthermore, the up-regulation of miR-1224-5p inhibits the proliferation and invasion and promotes the apoptosis of keloid cells via the TGF-β1/Smad3 signaling pathway (18). These data indicate that miR-1224-5p plays a tumor suppressor in malignant tumors. However, the function of miR-1224-5p and its potential molecular mechanisms in CRC remain unclear.

Epithelial–mesenchymal transition (EMT) is a process by which epithelial cells transform into invasive mesenchymal cell phenotypes and is involved in the invasion and metastasis of various cancer types (19, 20). A typical EMT is marked by a diminution of the cellular adhesion protein E-cadherin and a rise in the expression of N-cadherin and vimentin. E-cadherin is a key molecule in epithelial intercellular adhesion while vimentin and N-cadherin are markers for mesenchymal components (21). A growing number of research has shown that EMT is strongly associated with CRC invasion and metastasis (22, 23). However, little is known about the relationship between miR-1224-5p and EMT in CRC.

Our results show that the underexpression of miR-1224-5p is related to the poor prognosis of patients with CRC. miR-1224-5p exerts its function by directly targeting Sp1 transcription factor (SP1) to repress the migration, invasion, and EMT development of CRC cells *in vitro* and *in vivo*. Moreover, the analysis of clinical data indicates that miR-1224-5p, SP1, and their combination are significant for the prognostic prediction of patients with CRC.

## Materials and Methods

### Tissue Specimens

Eighty paired specimens (tumor and adjacent normal tissues) were obtained from CRC patients who accepted radical surgery at the Department of Colorectal Surgery, the First Affiliated Hospital of Nanjing Medical University (NJMU), China, in 2014–2015. All patients who received preoperative chemotherapy or radiotherapy were excluded. The diagnosis of pathology was confirmed by the Department of Pathology, the First Affiliated Hospital of Nanjing Medical University. The written informed consent of each patient or their relatives was obtained for the collection of specimens. All experiments were approved by the ethics committee of the First Affiliated Hospital of Nanjing Medical University. The study was conducted in accordance with the guidelines set by the Declaration of Helsinki. Samples were all collected within 5 min after resected, then immediately transferred to a −80°C freezer or embedded in paraffin.

### Cell Culture

The CRC cell lines, DLD1, HCT116, HT29, LOVO, SW480, and colon epithelium cells NCM460 were obtained from ATCC (Manassas, VA, USA). The cells were cultured in DMEM medium (Winsent, Canada) supplemented with 10% FBS (Gibco, CA, USA), 1% penicillin and streptomycin at 37°C. Cells were routinely examined for *Mycoplasma* contamination.

### Quantitative Real-Time PCR (qRT-PCR) and miRNA RT-PCR

Total RNA from CRC tissues and cells was extracted with TRIzol reagent (Invitrogen, USA) according to the manufacturer's instructions. Total RNA quality and quantity was measured by NanoDrop, and then reverse transcribed into cDNA through PrimeScript RT reagent kit (Takara, Dalian, China). cDNA was then amplified with a SYBR® Premix Ex Taq™ kit (Takara) using a 7500 Realtime PCR System (Applied Biosystems, Carlsbad, CA, USA). The results were calculated through relative quantification by the 2^−ΔΔCT^ method. The primers of the target mRNA and internal control were designed as follows: SP1 forward, 5′-TTGAAAAAGGAGTTGGTGGC-3′ and SP1 reverse, 5′-TGCTGGTTCTGTAAGTTGGG-3′; β-actin forward, 5′-CATGTACGTTGCTATCCAGGC-3′ and β-actin reverse, 5′-CTCCTTAATGTCACGCACGAT-3′. The specific Bulge-Loop^TM^ miRNA qRT-PCR primer for miR-1224-5p and U6 were designed by RiboBio (Guangzhou, China). All reactions were run in triplicate.

### Cell Transfection

Procedures were described previously (24). LV2-hsa-miR-1224-5p-mimic vector (miR-1224-5p-mimics), LV2-hsa-miR-1224-5p-inhibitor vector (miR-1224-5p-inhibitor), and LV2 empty lentivirus as a negative control (miR-NC) were constructed by lentiviral vectors (GenePharma, Shanghai, China). The cells were seeded in six-well plates and transfected at an appropriate multiplicity of infection. According to protocols, puromycin was used to screen the stable transfected cells. The SP1 overexpression plasmid and empty plasmid as negative control (NC) were designed and synthesized by GenePharma Inc. (Shanghai, China). Small interfering RNAs (siRNA) which for knocking down SP1 were obtained from RiboBio (Guangzhou, China).

### Western Blot Analysis

The RIPA kit (Beyotime, Shanghai, China) supplemented with phenylmethanesulfonyl fluoride was used to extract protein lysate from CRC tissues and cells according to the protocols. The BCA Protein Assay Kit (Beyotime, Shanghai, China) was used to quantify the protein concentration. The protein lysate was separated via 10% SDS-PAGE (Beyotime, Shanghai, China), next, it was transferred onto a polyvinylidene fluoride membrane (Millipore, USA). The membranes were blocked with 5% skim milk powder for 2 h and then incubated with the specific primary antibody overnight at 4°C, followed by a wash in TBST. Then, the membrane was incubated with the corresponding secondary antibody for 2 h at room temperature and then rinsed in TBST for three times (10 min each time). The level of protein expression was detected by ECL Plus (Millipore, USA) using a Bio-Imaging System (Bio-Rad, USA). The antibodies against SP1, IκBα, p- IκBα, p65, p-p65, E-cadherin, ZO-1, N-cadherin, Vimentin, HIF-1α, Histone H3, and β-actin were purchased from Abcam (Cambridge, MA, USA).

### Immunofluorescence

CRC cells transfected with corresponding miRNA vectors were seeded on glass bottom culture dishes, fixed with 4% paraformaldehyde for 20 min, and perforated with 0.1% Triton X-100 for 4 h at room temperature. After blocking with blocking buffer (Beyotime, Shanghai, China) for 1 h and washing by PBS, the cells were incubated with primary antibodies E-cadherin (1:100; Abcam) or Vimentin (1:250; Abcam) overnight at 4°C. The culture dishes were incubated with a AlexaFluor488-conjugated secondary antibodies (Beyotime, Shanghai, China) for 60 min at room temperature. The nuclei of the enterochromaffin cells were stained with DAPI (Beyotime, Shanghai, China) for 10 min at room temperature. Confocal fluorescence microscopy (Zeiss Germany, Germany) was used to capture fluorescence confocal images.

### Wound Healing Assay and Transwell Assays

Cells were seeded into six-well plates at 4 × 10^5^ cells/well until they reached almost 100% confluency, and a 200 μL pipette tip was used to scratch a linear wound. The medium was changed to serum-free DMEM at 0 and 24 h after wounding, and the migrating cells at the wound front were monitored by inverted microscopy.

Transwell inserts with and without Matrigel coating (8 μm pore size; Millipore) were used to perform cell migration and invasion. A total of 3 × 10^4^ cells were suspended in 200 μL of serum-free medium and added into the upper chamber, and 600 μL complete culture medium containing 20% FBS was added into the lower chamber. Cells were incubated for 24 h at 37°C. After fixed in 4% paraformaldehyde, cells were stained with crystal violet dye. A cotton swab was used to gently remove the cells on the inner chamber. Three fields of migrated and invaded cells were selected randomly and counted using light microscopy.

### Luciferase Report Assay

Report assay was conducted as previously described (24). The wild-type 3′-UTR sequence of SP1 predicted to interact with miR-1224-5p, referred to as WT-SP1 3′-UTR, together with a matched mutated sequence within potential binding sites, referred to as MUT-SP1 3′-UTR, were synthesized by GenScript (Nanjing, China). These sequences were inserted into the XbaI and SacI sites of the pmirGLO dual-luciferase miRNA target expression vector (Promega, USA). These vectors (WT-SP1 3′-UTR or MUT-SP1 3′-UTR together with miR-1224-5p-mimic/miR-1224-5p-inhibitor or miR-NC) were co-transfected into SW480 and HCT116 cells by using Lipofectamine 2000 reagent (Invitrogen, USA). Luciferase activity was measured with the Luciferase Reporter Assay System (Promega, USA) after transfection at 48 h.

### Immunohistochemistry

IHC was conducted as previously described (24). Tissue sections (thickness, 4 μm) were deparaffinized in xylene and rehydrated through graded alcohol. Endogenous peroxidase activity was blocked by incubation in 3% H_2_O_2_ for 10 min at room temperature. The specific antibodies (Abcam) were used as the primary antibodies by using a streptavidin peroxidase-conjugated method. And staining scores were observed in terms of staining intensity and percentage of positively stained cells. Staining intensity was divided into 4 grades: 0 (no staining), 1 (weak staining), 2 (intermediate staining), or 3 (strong staining). The staining percentage were divided into the following grades: 0 (<5% positive), 1 (<25% positive), 2 (25–50% positive), 3 for (51–75% positive), and 4 (>75% positive) (25). Ten independent fields (×400) were randomly selected to obtain an average score.

### *In vivo* Experiments

Twenty-four 5-week-old male BALB/c nude mice (23–25 g) were purchased from the Animal Core Facility of Nanjing Medical University, Nanjing, China. The mice were randomly divided into four groups: SW480-miR-1224-5p-inhibitor, SW480-miR-NC, HCT116-miR-1224-5p-mimics, and HCT116-miR-NC. The mice were anesthetized by inhalation of isoflurane (0.5–1.0%). The spleen was exteriorized through a vertical 1–1.5 cm subcostal incision in the left abdominal wall. Cells (1 × 10^6^ suspended in 20 μL phosphate buffered saline) were injected into the distal tip of the spleen with a Hamilton syringe. After 3 min, the spleen vessels were tied off using a suture, and the spleen was removed. The inner wound was closed with Vicryl-40 stitches, and the incision was closed with staples. Mice were sacrificed 6 weeks post-injection, and the liver was dissected and embedded in paraffin. The study was approved by the Institutional Animal Care and Use Committee of NJMU.

### Statistical Analysis

The data were shown as mean ± standard deviation. Each experiment was performed in triplicate independently. SPSS software 19.0 (Chicago, USA) and Graphpad Prism 8.0 (CA, USA) performed the analyses, including student *t*-test (two-tailed), Pearson's correlation analysis, Kaplan–Meier analysis, and log-rank test, based on the experimental design. The significance threshold was set at 0.05 in each test.

## Results

### Downregulated miR-1224-5p Expression Is Closely Related to the Metastasis of CRC

**Figure 1:**
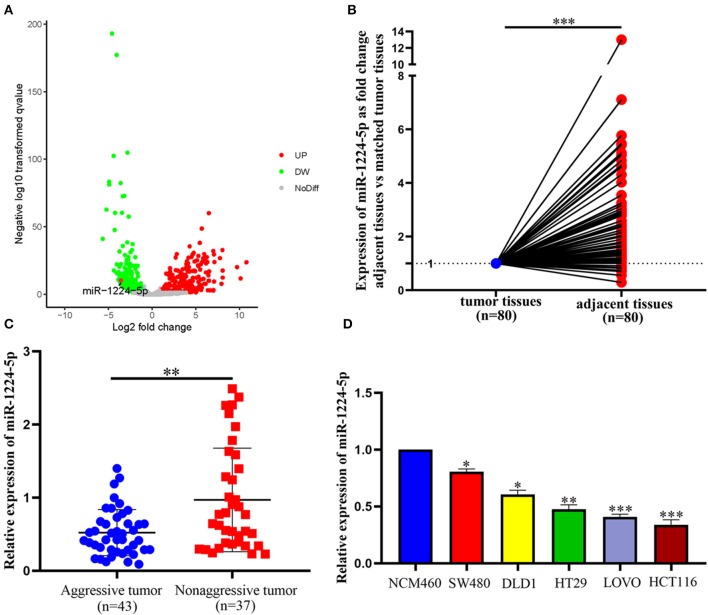
miR-1224-5p is underexpressed in CRC tissues and CRC cell lines. **(A)** miR-1224-5p expression in 457 CRC and 7 non-tumor colorectal tissues from TCGA data. **(B)** miR-1224-5p expression in 80 paired CRC tissues and adjacent normal tissues. **(C)** miR-1224-5p expression in aggressive and non-aggressive CRC tissues. **(D)** miR-1224-5p expression in CRC cell lines and normal epithelial colon cell NCM460. Data represent the mean ± SD from three independent experiments. Student's *t*-test was used to determine statistical significance: *p < 0.05, **p < 0.01, and ***p < 0.001.

The expression of miR-1224-5p was more dramatically downregulated in 457 CRC samples than in 7 non-tumor colorectal tissues from TCGA data (Figure 1A). In addition, the low expression of miR-1224-5p was conferred in 80 paired CRC tissues and adjacent normal tissues by qRT-PCR (Figure 1B). We defined the CRC tissues with distant metastasis, lymph node metastasis, and tumor invasion via nerve or venous infiltration as aggressive CRC tissues. Figure 1C shows that the level of miR-1224-5p expression in an aggressive tumor was lower than that in a non-aggressive tumor. The level of miR-1224-5p expression in CRC cell lines and NCM460 were also detected. As expected, the normal epithelial colon cell NCM460 showed a higher level of miR-1224-5p expression compared with the CRC cell lines (Figure 1D). To further investigate the association between miR-1224-5p expression levels and clinical characteristics, this study divided the patients into the high expression group and low expression group on the basis of the expression levels of miR-1224-5p, respectively. The result demonstrates that the expression of miR-1224-5p is inversely related to tumor metastasis (Table 1).

**Table 1:** Expression of miR-1224-5p and SP1 according to patients' clinical features.

**Characteristics**	**Number**	**miR-1224-5p expression**		***P*-value**	**SP1 expression**		***P*-value**
		**High group**	**Low group**		**High group**	**Low group**	
**Age (years)**							
<50	16	7	9	0.58	6	10	0.26
>50	64	33	31		34	30	
Gender							
Female	48	26	22	0.36	23	25	0.65
Male	32	14	18		17	15	
**Lymph node metastasis**							
No	47	30	17	0.003^**^	16	31	<0.001^***^
Yes	33	10	23		24	9	
Tumor stage							
Stage I, II	37	25	12	0.004^**^	10	27	<0.001^***^
Stage III, IV	43	15	28		30	13	
**TNM staging system**							
T1+T2	45	30	15	<0.001^***^	13	32	<0.001^***^
T3+T4	35	10	25		27	8	
**Distant metastasis**							
No	62	35	27	0.03^*^	26	36	0.007^**^
Yes	18	5	13		14	4	
**Tumor size(cm)**							
<3 cm	48	23	25	0.65	20	28	0.06
>3 cm	32	17	15		20	12	

* *P < 0.05*,

** P < 0.01, and

*** *P < 0.001*.

### miR-1224-5p Inhibits CRC Cell Migration and Invasion *In vitro*

**Figure 2:**
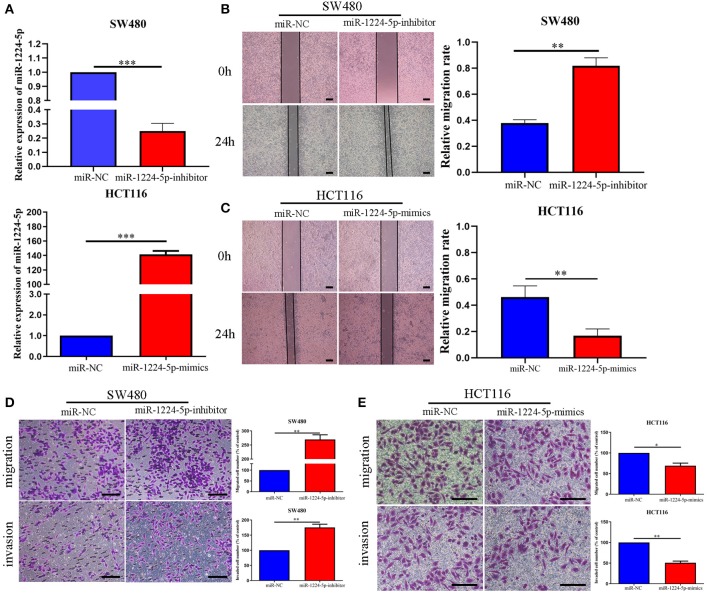
miR-1224-5p inhibits the migration and invasion of CRC cells *in vitro*. **(A)** SW480 and HCT116 cells that were transfected with miR-1224-5p-inhibitor and miR-1224-5p-mimics lentivirus were subjected to qRT-PCR for miR-1224-5p. **(B,C)** Effects of miR-1224-5p on migration in CRC cell lines were detected by wound healing assay, scale bars: 300 μm. **(D)**. Cell migration and invasion as measured by Transwell assays were promoted by miR-1224-5p knockdown in SW480 cells, scale bars: 100 μm. **(E)** miR-1224-5p overexpression inhibited migration and invasion of HCT116 cells, scale bars: 50 μm. Data represent the mean ± SD from three independent experiments. Student's *t*-test was used to determine statistical significance: *p < 0.05, **p < 0.01, and ***p < 0.001.

We selected two CRC cell lines, SW480 and HCT116, to transfect the miR-1224-5p-inhibitor and mimics lentivirus via the level of miR-1224-5p expression and investigate the biological function of miR-1224-5p in CRC, respectively. The transfection efficiency depicted in Figure 2A confirms that miR-1224-5p was knocked down in SW480 cells and overly overexpressed in HCT116 cells by qRT-PCR. First, we assessed cell migration and demonstrated that downregulated miR-1224-5p increased the migration of SW480 via wound healing assays, whereas miR-1224-5p overexpression greatly inhibited the migration of HCT116 cells (Figures 2B,C). Transwell assays indicated that the downregulated miR-1224-5p increased the migration and invasion of SW480 cells relative to the control group (Figure 2D). By contrast, the HCT116 cells overexpressed miR-1224-5p revealed a substantial decrease in cell migration and invasion (Figure 2E). Moreover, we evaluated cell proliferation by CCK-8 assays and EdU incorporation and there was no significance after regulating the level of miR-1224-5p expression in CRC cells (Figures S1A,B). These findings suggest that upregulated miR-1224-5p inhibits the migration and invasion of CRC cell.

### miR-1224-5p Regulates the EMT of CRC Cells

**Figure 3:**
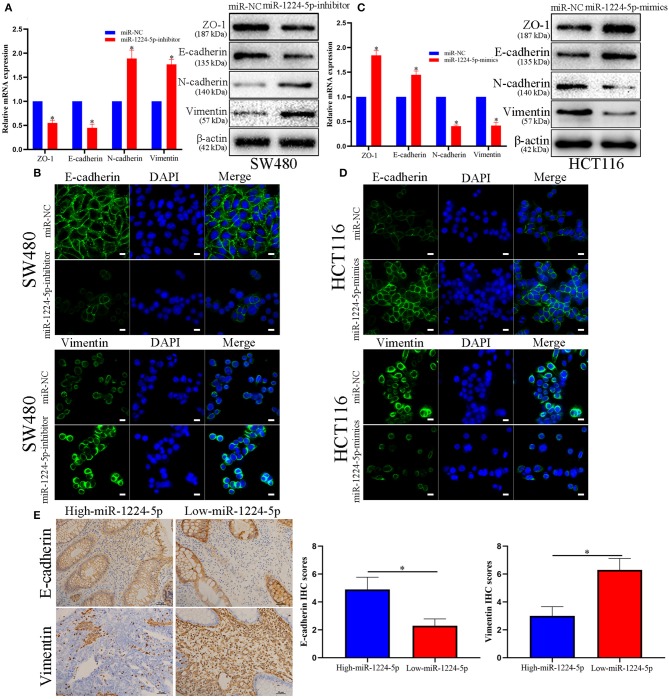
miR-1224-5p inhibits the EMT process of CRC cells. **(A)** Western blot analysis of tight junction protein ZO-1, epithelial marker E-cadherin, and mesenchymal markers N-cadherin and Vimentin after miR-1224-5p knockdown in SW480 cells. **(B)** IF staining of E-cadherin and Vimentin after miR-1224-5p knockdown in SW480 cells, scale bars: 10 μm. **(C)** miR-1224-5p overexpression increased ZO-1 and E-cadherin expression, and decreased the levels of N-cadherin and Vimentin in HCT116 cells. **(D)** IF staining of E-cadherin and Vimentin after miR-1224-5p overexpression in HCT116 cells, scale bars: 10 μm. **(E)** Immunohistochemistry of E-cadherin and Vimentin were detected and compared between miR-1224-5p high expressing CRC tissues and miR-1224-5p low expressing cases. Data represent the mean ± SD from three independent experiments. Student's *t*-test was used to determine statistical significance: *p < 0.05.

EMT plays an important role in the regulation of metastasis in CRC (26). Further research was performed to verify whether miR-1224-5p inhibits CRC metastasis by modulating EMT. We used qRT-PCR and Western blot analysis to evaluate the roles of abnormal miR-1224-5p in EMT. The results of Figure 3A show that downregulated miR-1224-5p in SW480 decreased the expression of tight junction protein ZO-1 and epithelial marker E-cadherin and increased the expression of the mesenchymal markers N-cadherin and vimentin in mRNA and protein levels, respectively. Conversely, overexpressed miR-1224-5p presented a substantial increase in the expression of E-cadherin and ZO1 and decreased the levels of vimentin and N-cadherin expression in HCT116 (Figure 3C). Furthermore, we also determined the transcription factors of EMT including ZEB-1, Snail and Twist and observed that downregulated miR-1224-5p in SW480 increased the expression of ZEB-1, Snail and Twist in mRNA and protein levels, and overexpressed miR-1224-5p decreased in the expression of ZEB-1, Snail and Twist in HCT116 (Figures S2A,B). The results of immunofluorescence show that downregulated miR-1224-5p promotes EMT progression by reducing E-cadherin expression and increasing the vimentin quantity of SW480, whereas upregulated miR-1224-5p produces the opposite effects in HCT116 (Figures 3B,D). In addition, we further explored the correlation between the expression of miR-1224-5p and EMT markers in CRC tissues. We found that E-cadherin expression in miR-1224-5p high expressing CRC tissues was notably higher than that in low expressing cases. Correspondingly, the level of Vimentin expression in the low-expression group of mir-1224-5p was significantly higher than that in the high-expression group (Figure 3E). These findings demonstrate that miR-1224-5p suppresses the EMT of CRC cells.

### SP1 Is the Direct Target of miR-1224-5p

**Figure 4:**
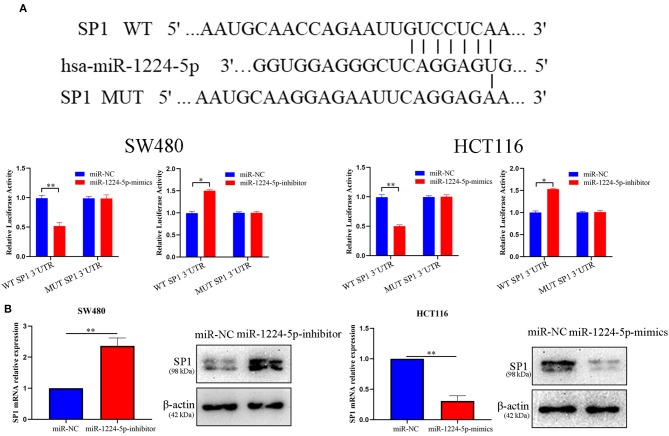
SP1 was proved to be a potential target of miR-1224-5p. **(A)** Luciferase reporter assay was conducted to verify that miR-1224-5p bound to the 3′-UTR region of SP1 directly. miR-1224-5p overexpression significantly suppressed, while miR-1224-5p loss increased the luciferase activity that carried wild-type (wt) but not mutant (mut) 3′-UTR of SP1. **(B)** miR-1224-5p knockdown increased the expression of SP1 mRNA and protein in SW480 cells and miR-1224-5p overexpression decreased the level of SP1 mRNA and protein in HCT116 cells, respectively. Data represent the mean ± SD from three independent experiments. Student's *t*-test was used to determine statistical significance: *p < 0.05 and **p < 0.01.

**Figure 5:**
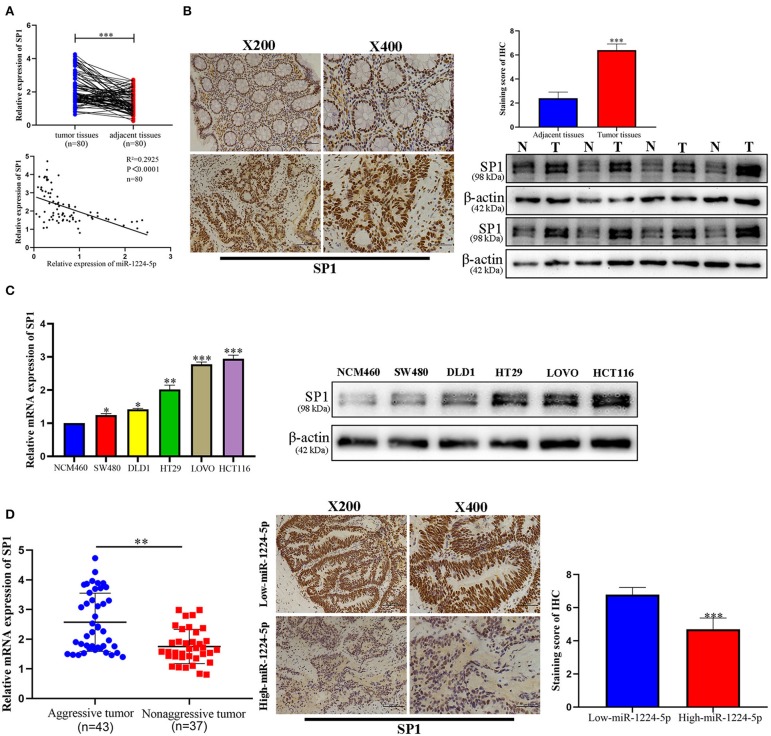
Expression pattern of SP1 in CRC. **(A)** Expression of SP1 was upregulated in 80 paired CRC tissues compared with adjacent normal tissues and Pearson's correlation analysis showed the negative correlation of SP1 with miR-1224-5p. **(B)** Immunohistochemistry showed the expression level of SP1 in CRC tissues and paired adjacent normal tissues, western blot showed the expression level of SP1 protein in 8 paired CRC tissues and adjacent normal tissues. **(C)** Expression of SP1 mRNA and protein in CRC cell lines and normal epithelial colon cell NCM460 were detected by qRT-PCR and western blot. **(D)** The mRNA expression of SP1 in aggressive tumors was significantly higher than that in non-aggressive tumors, as determined by qRT-PCR. Representative immunohistochemical staining showed a weak staining of SP1 in miR-1224-5p high-expressing CRC tissue and strong staining of SP1 in the miR-1224-5p low-expressing tumor. Data represent the mean ± SD from three independent experiments. Student's *t*-test was used to determine statistical significance: *p < 0.05, **p < 0.01, and ***p < 0.001.

To illustrate the specific mechanism by which miR-1224-5p exerts its biological effects in CRC, we predicted the potential targets by using bioinformatic tools in public databases, including miRanda and TargetScan. We found a conserved putative miR-1224-5p bound to the 3′-UTR of SP1 mRNA. To validate this hypothesis, luciferase reporter assay was performed and the results confirmed that miR-1224-5p directly bind to 3′-UTR of SP1 mRNA (Figure 4A). Next, qRT-PCR and Western blot analysis confirmed that the downregulated miR-1224-5p increased and that the overexpressed miR-1224-5p decreased the expression of SP1 mRNA and protein in the CRC cells (Figure 4B). We detected the expression of SP1 in CRC tissues and cell lines and its correlation with miR-1224-5p. The level of SP1 expression was more upregulated in the CRC tissues compared to adjacent normal tissues and Pearson's correlation analysis found that the SP1 was negatively correlated with miR-1224-5p (Figure 5A). IHC and Western blot analysis were performed to detect the expression of SP1 in eight paired CRC tissues and adjacent normal tissues (Figure 5B). In order to investigate the association between expression patterns and clinical features, and we discovered that high expression levels of SP1 are closely related with CRC metastasis (Table 1). In addition, the level of SP1 mRNA and protein was increased in the CRC cell lines but not in the NCM460 (Figure 5C). Figure 5D shows that the expression level of SP1 in an aggressive tumor was higher than that in a non-aggressive tumor. Moreover, we found via IHC that the expressions of SP1 in the miR-1224-5p high-expressing tumors were substantially lower than those in the miR-1224-5p low-expressing tumors (Figure 5D). Overall, these data indicate that miR-1224-5p can directly negatively regulate SP1.

### miR-1224-5p Inhibits Migration and Invasion by Targeting SP1

**Figure 6:**
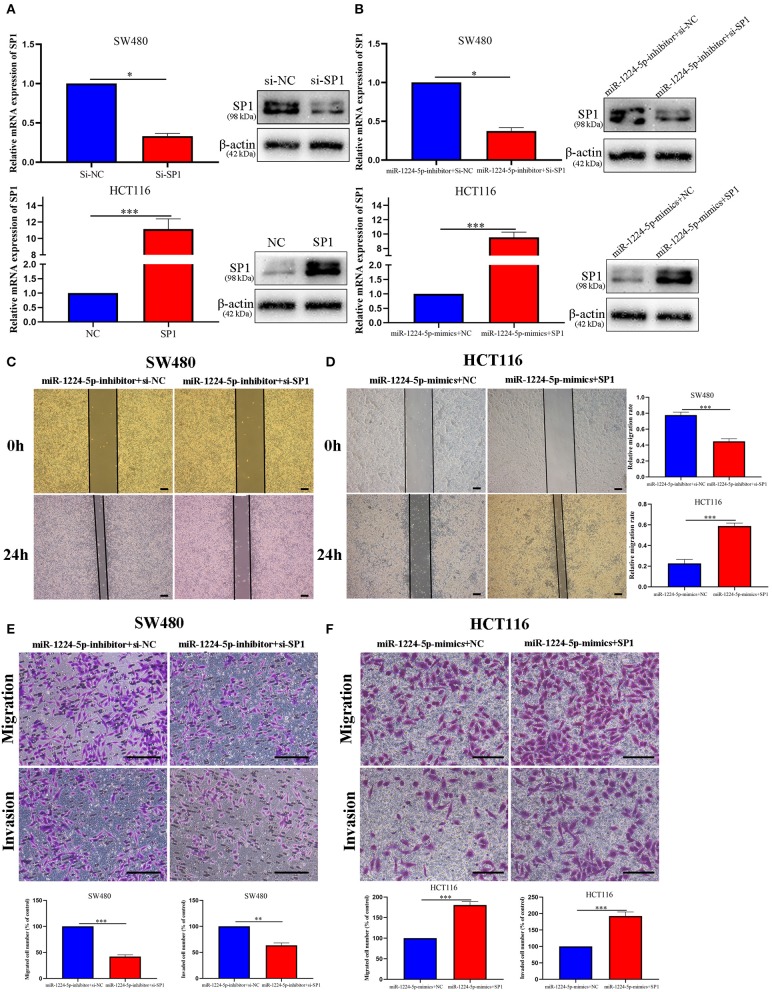
MiR-1224-5p inhibits migration and invasion by targeting SP1. **(A)** Expression of SP1 mRNA and protein was verified in transfected CRC cell lines by qRT-PCR and western blot. **(B)** Expression of SP1 was confirmed by qRT-PCR and western blot in co-transfected CRC cell lines. **(C,E)** Wound healing assay and Transwell assays confirmed that SP1 knockdown abrogated the effects of miR-1224-5p loss on migration and invasion of SW480 cells, scale bars: 300 μm **(C)** and 100 μm **(E)**. **(D,F)** SP1 restoration promoted migration and invasion of miR-1224-5p-overexpressing of HCT116 cells, scale bars: 300 μm **(D)** and 50 μm **(F)**. Data represent the mean ± SD from three independent experiments. Student's *t*-test was used to determine statistical significance: *p < 0.05, **p < 0.01, and ***p < 0.001.

In order to evaluate the biological role of SP1 interacting with miR-1224-5p in CRC, we performed the rescue experiments. First, we used siRNA-SP1 and pcDNA3.1-SP1 to downregulate or upregulate SP1 in SW480 and HCT116 cells, respectively. We detected the expression of SP1 via qRT-PCR and Western blot analysis (Figure 6A). In SW480 cells, co-downregulated miR-1224-5p and SP1 cells showed a lower expression of SP1 than the downregulated miR-1224-5p. and in HCT116, co-upregulated miR-1224-5p and SP1 cells showed a higher expression of SP1 than the upregulated miR-1224-5p group (Figure 6B). Subsequently, the wound healing assay indicated that the SP1 knockdown in miR-1224-5p-inhibitor SW480 cells substantially reversed the promotion function induced by miR-1224-5p loss on the migration of SW480 cells (Figure 6C). SP1 restoration eliminated the inhibitory effects of miR-1224-5p on HCT116 cell migration (Figure 6D). Similarly, Transwell assay confirmed that the effects of promoting SW480 migration and invasion made by downregulated miR-1224-5p can be counteracted by downregulated SP1 (Figure 6E). Upregulated SP1 abolished the effects of upregulated miR-1224-5p in suppressing HCT116 cell migration and invasion, as shown in Figure 6F. These findings suggest that miR-1224-5p inhibits metastasis in CRC by targeting SP1 expression.

### MiR-1224-5p Inhibits the Cell Metastasis of CRC *In vivo*

**Figure 7:**
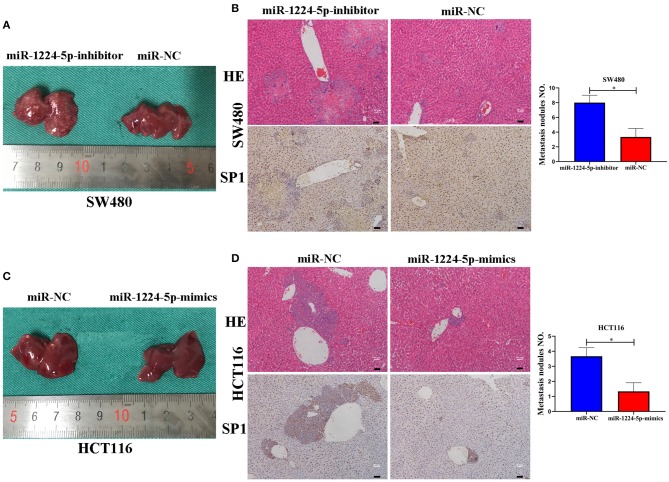
miR-1224-5p inhibits the liver metastasis of CRC cells *in vivo*. **(A,C)**. Representative photograph of liver metastases obtained from nude mice transfected with miR-1224-5p-inhibitor, miR-1224-5p-mimics, and miR-NC. **(B,D)**. HE staining and immunohistochemistry of liver metastases in miR-1224-5p-inhibitor, miR-1224-5p-mimics, and miR-NC, scale bars: 50 μm **(B,D)**. Data represent the mean ± SD from three independent experiments. Student's *t*-test was used to determine statistical significance: *p < 0.05.

In order to reveal the biological effects of miR-1224-5p *in vivo*, we injected downregulated miR-1224-5p SW480 cells (SW480-miR-1224-5p-inhibitor) and control cells (SW480-miR-NC) or overexpressed miR-1224-5p HCT116 cells (HCT116-miR-1224-5p-mimics) and control cells (HCT116-miR-NC) into the distal tip of the spleen of nude mice. The miR-1224-5p knockdown group showed the most number of and largest foci in the liver of nude mice (Figure 7A). We also confirmed that the liver sections of the miR-1224-5p knockdown group showed increased expression of SP1 (Figure 7B). By contrast, overexpressed miR-1224-5p in HCT116 cells led to a substantial decrease in liver metastasis nodules and SP1 expression (Figures 7C,D). These findings demonstrate that miR-1224-5p represses the metastatic behaviors of CRC and regulates the level of SP1 expression *in vivo*.

### NF-κB Signaling Is Essential for the Biological Function of miR-1224-5p in CRC Cells

**Figure 8:**
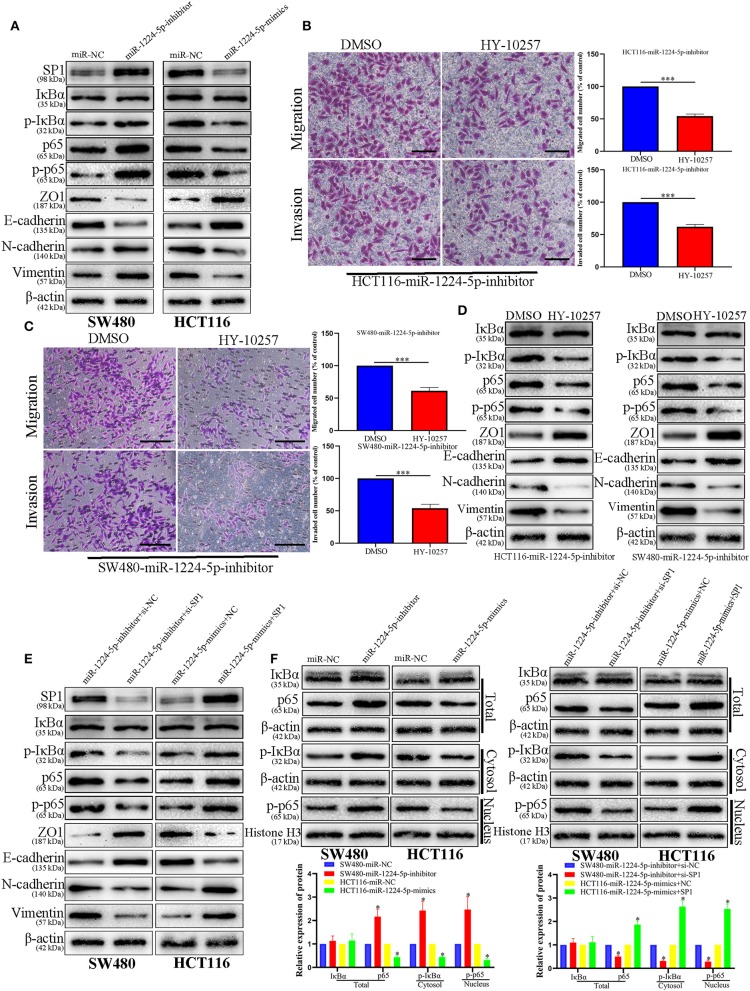
NF-κB signaling is essential for the biological function of miR-1224-5p in CRC. **(A)** SW480 and HCT116 cells that were transfected with corresponding miRNA lentivirus were subjected to immunoblotting for SP1, IκBα, phosphorylated IκBα, p65, phosphorylated p65, and EMT. **(B,C)** NF-κB inhibitor HY-10257 treatment abrogated the effect of miR-1224-5p loss on mobility of HCT116 and SW480 cells, scale bars: 50 μm **(B)** 100 μm **(C,D)** Western blot analysis indicated that modulating p65 phosphorylation reversed the effects of miR-1224-5p alteration on EMT process of CRC cells. **(E)** Expression of protein in co-transfected and transfected CRC cells was detected by western blot. **(F)** IκBα, phosphorylated IκBα, p65 and phosphorylated p65 protein levels in whole cell lysate, cytosol and nucleus were analyzed by Western blot. Data represent the mean ± SD from three independent experiments. Student's *t*-test was used to determine statistical significance: *p < 0.05, ***p < 0.001.

Prevenient researches reported that SP1 participates in the biological function of NF-κB signaling pathways and EMT (27). Transfected SW480 and HCT116 cells were subjected to immunoblotting for SP1, IκBα, phosphorylated IκBα, p65, phosphorylated p65, and EMT proteins (ZO1, E-cadherin, vimentin, and N-cadherin). The results showed that the downregulated miR-1224-5p promoted the phosphorylation of p65 and facilitated EMT, whereas the overexpressed miR-1224-5p decreased the level of phosphorylated p65 and inhibited EMT in CRC cells (Figures 8A–F). These results show that miR-1224-5p suppresses the activation of NF-κB pathways in CRC cells. We confirmed such effect by repressing SP1 in SW480 and upregulating SP1 in HCT116 (Figures 8E,F). Downregulated miR-1224-5p HCT 116 and SW480 cells showed deterred migration and invasion after treatment with NF-κB pathway inhibitor HY-10257 (Figures 8B,C). Western blot analysis revealed the same results for HY-10257 causing the inhibition of NF-κB pathways and EMT in SW480 and HCT116 cells transfected with corresponding miRNA lentivirus (Figure 8D).

### Clinical Significance of miR-1224-5p and SP1 Expression in Patients With CRC

**Figure 9:**
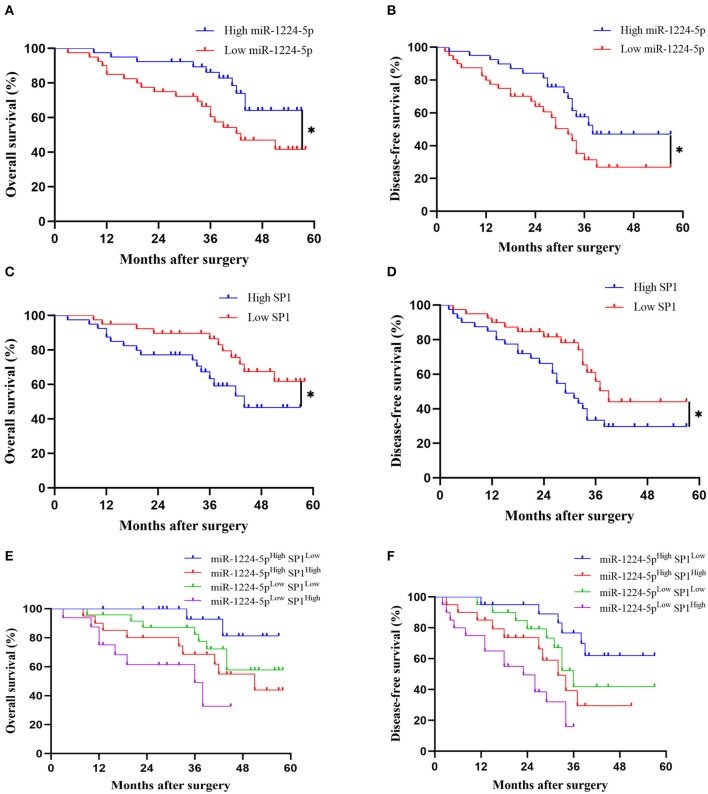
The prognostic value of miR-1224-5p and SP1 in patients with CRC. **(A,B)** Comparison of overall survival (OS) and disease-free survival (DFS) between patients with high expression of miR-1224-5p and low expressing cases in CRC via Kaplan–Meier analysis. **(C,D)**. Comparison of OS and DFS between patients with high expression of SP1 and low expressing cases in CRC. **(E,F)**. Comparison of OS and DFS between four subgroups of CRC patients (subgroup I: high miR-1224-5p and low SP1; subgroup II: high miR-1224-5p and high SP1; subgroup III: low miR-1224-5p and low SP1; subgroup IV: low miR-1224-5p and high SP1). Log-rank test was used to determine statistical significance: *p < 0.05.

Kaplan–Meier survival curves showed a substantial reduction in the overall survival (OS) and disease-free survival (DFS) of patients with CRC with low expression of miR-1224-5p (Figures 9A,B), whereas patients with high expressions of SP1 showed substantially worse OS and DFS (Figures 9C,D). Further combinatorial analysis revealed that the patients with low expression of miR-1224-5p and high expression of SP1 had the worst OS and DFS (Figures 9E,F). Aforementioned results indicate that the combination of miR-1224-5p and SP1 can be a potential effective biomarker for clinical prognosis in patients with CRC.

### miR-1224-5p Reverses the Facilitation of Hypoxia on Metastasis and EMT of CRC Cells

**Figure 10:**
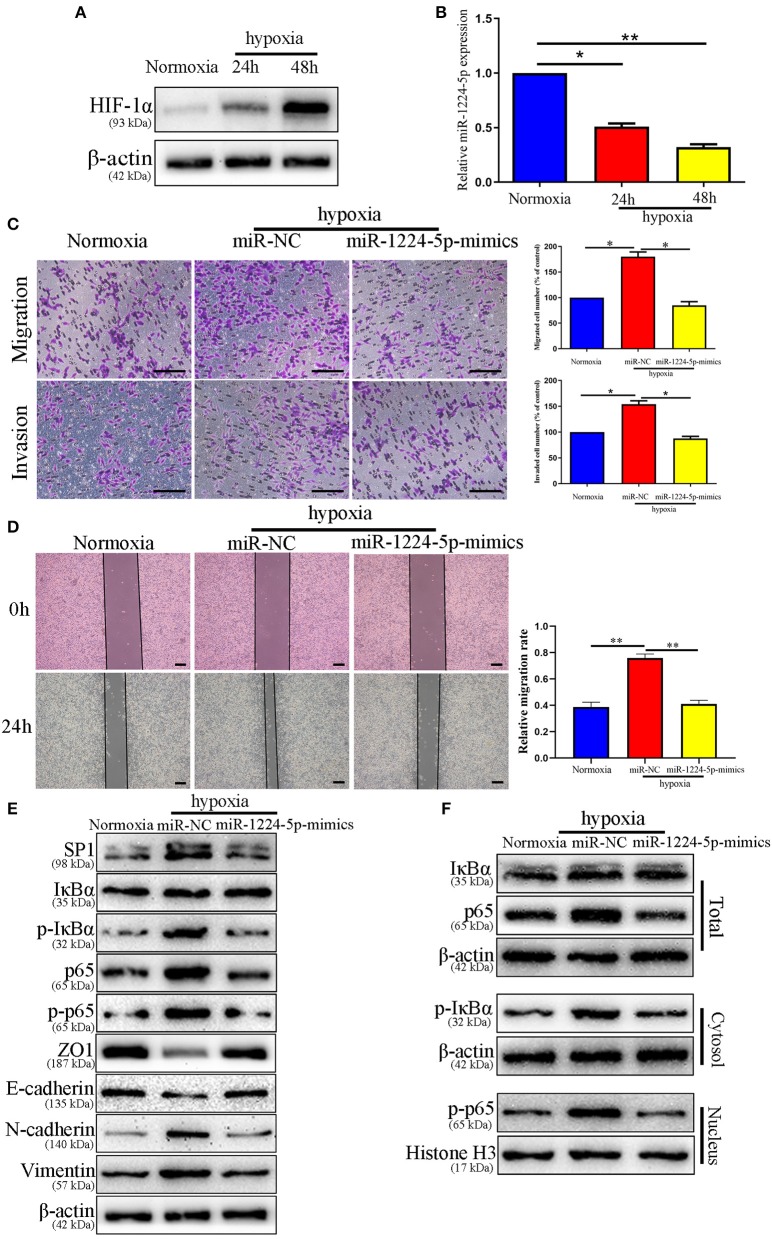
miR-1224-5p mediates the promoting effects of hypoxia on metastasis and EMT of CRC cells. **(A)** The expressions of HIF-1α in different time points in normoxia and hypoxia condition. **(B)** The levels of miR-1224-5p in SW480 cells cultured in normoxia and hypoxia. **(C,D)** Transwell assays and Wound healing assay revealed that hypoxia promoted migration and invasion of SW480 cells, while miR-1224-5p overexpression abolished the effects of hypoxia, scale bars: 100 μm **(C)** and 300 μm **(D,E)**. Hypoxia facilitated the EMT process of SW480 cells and miR-1224-5p restoration showed an opposite effect. **(F)** IκBα, phosphorylated IκBα, p65, and phosphorylated p65 protein levels in whole cell lysate, cytosol and nucleus were analyzed by Western blot. Data represent the mean ± SD from three independent experiments. Student's *t*-test was used to determine statistical significance: *p < 0.05, and **p < 0.01.

Hypoxia is the main feature of cancer microenvironments (23). To induce hypoxic conditions (<1% O_2_), we cultured SW480 cells at 37°C in an AnaeroPack™ jar with AnaeroPack™-Anaero (Mitsubishi, Japan) according to the manufacturer's instruction. The effect of hypoxia was evaluated by the expression of HIF-1α after 24 and 48 h of hypoxia treatment (Figure 10A). The relative expressions of miR-1224-5p in the SW480 cells declined after hypoxia treatment in a time-dependent manner (Figure 10B). The migration and invasion of the SW480 cells after hypoxia treatment were greatly enhanced, whereas this increased metastasis was rescued in overexpressed miR-1224-5p SW480 cells (Figures 10C,D). The data of the Western blot analysis demonstrated that overexpressed miR-1224-5p rescued the hypoxia-induced EMT progression of the decreased expressions of SP1, p- IκBα, p-p65, N-cadherin, and vimentin and the increased levels of ZO1 and E-cadherin in SW480 cells (Figures 10E,F). These findings demonstrate that miR-1224-5p reverses the promotion of hypoxia in the metastasis and EMT of CRC cells.

## Discussion

Considerable research has shown that aberrantly expressed miRNAs are closely related to the initiation and progression of CRC (22, 28). In addition, miRNAs are novel prognostic biomarkers and potential therapeutic targets for CRC (29). For the first time, this study found that miR-1224-5p is remarkably downregulated in CRC tissues and cell lines. Furthermore, the aggressive phenotype of CRC presented a lower level of miR-1224-5p expression than non-aggressive tumor. In addition, we found that HCT116 showed the higher metastatic ability with the lower expression level of miR-1224-5p compared with SW480. These data show that miR-1224-5p exerts a tumor suppressor in CRC.

Distant and local metastases are the leading causes of poor prognosis in patients with CRC. Increasing evidence suggests that miRNAs play a key role in regulating the metastasis of cancer, including CRC (30). In the present study, gain- and loss-of-function experiments verified that overexpressed miR-1224-5p inhibits the migration and invasion of CRC cells, whereas down-regulated miR-1224-5p promotes the metastatic behaviors of CRC cells. EMT is a key process in regulating the progression of metastasis in CRC (31). In this research, we investigated that upregulated miR-1224-5p inhibits EMT in CRC cells. We also detected that miR-1224-5p overexpressing CRC cells show upregulated E-cadherin and downregulated vimentin. These data indicate that miR-1224-5p inhibits CRC metastatic behaviors by repressing EMT.

In order to investigate the specific mechanism of miR-1224-5p in CRC, bioinformatic tools were used to find the putative targets of miR-1224-5p. We chose to study SP1 through preliminary validation in CRC tissues via qRT**-**PCR, which is dysregulated in different cancers (27, 32, 33). Moreover, SP1 acts a critical part in the EMT of CRC (27, 34). Then we demonstrated that miR-1224-5p directly targets SP1 and exercises its biological function in CRC. First, miR-1224-5p represses SP1 abundance in CRC cells. Second, the complementary base of miR-1224-5p is found in the 3′UTR of SP1 mRNA. The luciferase activity of WT 3′UTR of SP1, and not that of MUT 3′UTR, is changed by the overexpression and knocking down of miR-1224-5p. Third, SP1 is negatively correlated with miR-1224-5p expression in CRC tissues. SP1 mediates the miR-1224-5p regulation of CRC cell migration, invasion, and EMT. Previous studies have demonstrated that SP1 is involved in the up-regulation of p65 (NF-κB subunit) through binding to its promoter region and then enhancing its transcriptional activity (27, 35, 36). We first investigated whether SP1 is directly targeted by miR-1224-5p to verify whether SP1 participating in up-regulation of p65 expression. We found that the overexpression of miR-1224-5p significantly inhibited the expression of SP1, thereby reducing the expression of p65. The overexpression of SP1 abolished the changes of mir-1224-5p-induced EMT phenotype and related EMT markers. To further verify whether the activation of NF-κB is dependent on SP1 expression in miR-1224-5p-induced EMT, the expression levels of p-IκB, and p-p65 in miR-1224-5p-induced CRC cells were blocked when we decreased the expression of SP1. ZEB1 and TWIST1, which are the major transcription factors and regulators in the EMT, are reported to be regulated by NF-κB (37, 38). In addition, the expression of Vimentin is directly regulated by NF-κB through binding to its promoter region (39). The activation of the NF-κB signaling pathway is related to the malignant transformation and progression of CRC and the regulation of malignant characteristics of cancer cells (40, 41). In addition, NF-κB plays a critical role in CRC EMT (42). Here, we demonstrated that NF-κB inhibitors terminate the stimulation of miR-1224-5p knockdown during CRC cell migration, invasion, and EMT. Therefore, the role of miR-1224-5p in CRC cells might be associated with the SP1/NF-κB pathway.

Whether miR-1224-5p and SP1 are valuable predictors for the diagnosis and prognosis of patients with CRC must be proven. We discovered that the low-expression of miR-1224-5p and high-expression of SP1 are remarkably related to unfavorable clinical features in patients with CRC. Furthermore, low levels of miR-1224-5p and overexpression of SP1 and their combination are significantly related with poor prognosis in patients with CRC. Abovementioned results indicate that miR-1224-5p and SP1 may be promising biomarkers to predict prognosis in patients with CRC.

**Figure 11:**
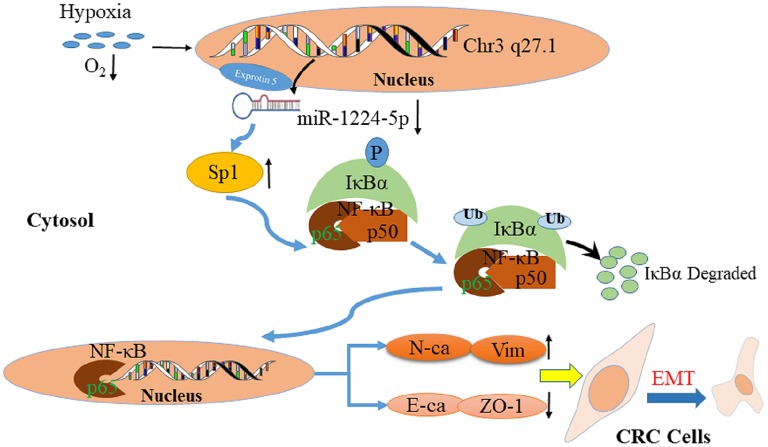
Proposed model of miR-1224-5p-induced EMT via targeting SP1-mediated NF-κB signaling pathway in CRC cells.

Previous studies confirmed that a hypoxic environment leads to the abnormal expression of miRNAs and promotes CRC metastasis (43, 44). Therefore, we attempted to determine the association between hypoxic environment and miR-1224-5p in CRC. A substantial drop of miR-1224-5p expression was observed after hypoxia treatment. In addition, overexpressed miR-1224-5p reversed the stimulation of hypoxia during the metastasis and EMT of CRC cells. Abovementioned data indicate that hypoxia-induced miR-1224-5p depletion promotes the migration, invasion and EMT of CRC (Figure 11).

Taken together, this study firstly confirms that the level of miR-1224-5p decreases in both CRC tissues and cell lines and that decreased the level of miR-1224-5p expression is associated with advanced clinicopathological features. Moreover, miR-1224-5p inhibits the metastasis and EMT of CRC cells by directly targeting the SP1-mediated NF-κB pathway. miR-1224-5p under expression, SP1 over expression, and the combination of them are promising prognostic biomarker for CRC patients. In addition, hypoxia is one of the key factors of miR-1224-5p reduction in CRC cells. In conclusion, the abnormal of miR-1224-5p act a vital role in tumor migration and invasion and may be a novel biomarker of prognosis and promising target of therapy for CRC.

## Data Availability Statement

All datasets generated for this study are included in the article/Supplementary Material.

## Ethics Statement

The studies involving human participants were reviewed and approved by the Ethics Committee of the First Affiliated Hospital of Nanjing Medical University. The patients/participants provided their written informed consent to participate in this study. The animal study was reviewed and approved by the Animal Ethics Committee of Nanjing Medical University.

## Author Contributions

JL, WP, PY, and RC designed and performed the experiments. WQ, QG, DJ, QW, and ZZ interpreted the data. JL, JT, and YS wrote the manuscript. YS and JT supervised the overall research, secured funding, and interpreted results.

### Conflict of Interest

The authors declare that the research was conducted in the absence of any commercial or financial relationships that could be construed as a potential conflict of interest.

## Supplementary Material

Figure S1 miR-1224-5p does not regulate the cell growth in CRC cells. **(A)** CCK-8 was used to detect the cell growth after modulating miR-1224-5p expression in CRC cells. **(B)** Effects of miR-1224-5p on proliferation in CRC cell lines were detected by EdU, scale bars: 50 μm. Student's *t*-test and log-rank test were used to determine statistical significance.

Figure S2 miR-1224-5p suppresses EMT process of CRC cells. **(A)** SW480 cells that were transfected with miR-1224-5p-inhibitor and miR-NC, respectively, were subjected to qRT-PCR and immunoblotting for the expression of EMT-related markers including ZEB-1, Snail and Twist. **(B)** Overexpressed miR-1224-5p decreased the levels of ZEB-1, Snail, and Twist in HCT116 cells. Data represent the mean ± SD from three independent experiments. Student's *t*-test was used to determine statistical significance: ^*^*P* < 0.05.

## Abbreviations

3′-UTR: 3′-untranslated region
CRC: colorectal cancer
SP1: Sp1 transcription factor
EMT: epithelial-mesenchymal transition
HE: hematoxylin and eosin
IF: immunofluorescence
IHC: immunohistochemistry
miRNAs: microRNAs
qRT-PCR: quantitative real-time polymerase chain reaction
RT-PCR: reverse transcription polymerase chain reaction
TNM: tumor-node-metastasis
OS: overall survival
DFS: disease-free survival.
