# Supplementary figures and images for: MicroRNA-1224-5p Inhibits Metastasis and Epithelial-Mesenchymal Transition in Colorectal Cancer by Targeting SP1-Mediated NF-κB Signaling Pathways
Source: Front Oncol. 2020 Mar 13;10:294. doi: 10.3389/fonc.2020.00294 (PMC7083241; doi:10.3389/fonc.2020.00294)

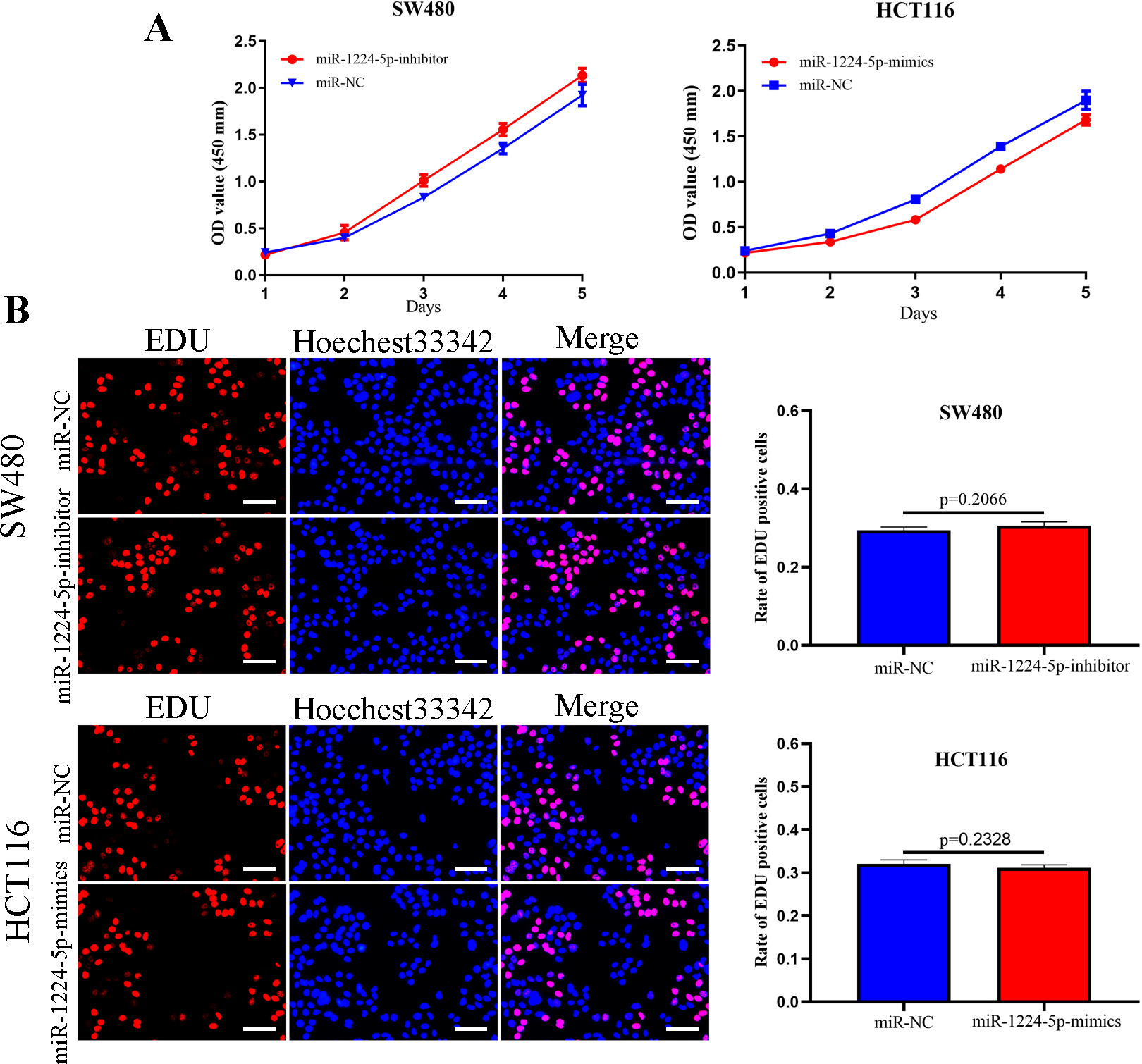

Supplement: Figure S1 — miR-1224-5p does not regulate the cell growth in CRC cells. (A) CCK-8 was used to detect the cell growth after modulating miR-1224-5p expression in CRC cells. (B) Effects of miR-1224-5p on proliferation in CRC cell lines were detected by EdU, scale bars: 50 μm. Student's t-test and log-rank test were used to determine statistical significance. [file Image_1.TIF]

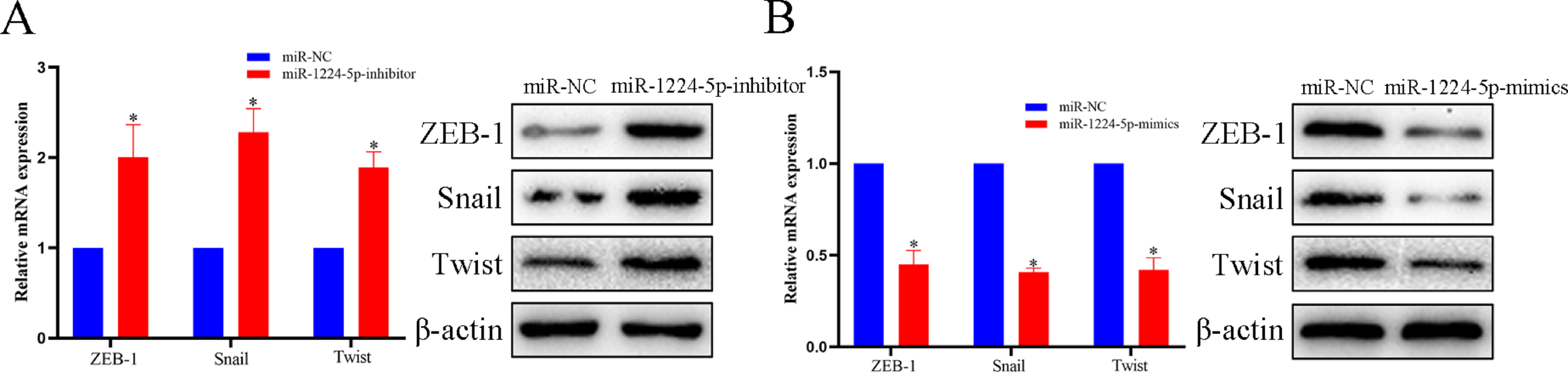

Supplement: Figure S2 — miR-1224-5p suppresses EMT process of CRC cells. (A) SW480 cells that were transfected with miR-1224-5p-inhibitor and miR-NC, respectively, were subjected to qRT-PCR and immunoblotting for the expression of EMT-related markers including ZEB-1, Snail and Twist. (B) Overexpressed miR-1224-5p decreased the levels of ZEB-1, Snail, and Twist in HCT116 cells. Data represent the mean ± SD from three independent experiments. Student's t-test was used to determine statistical significance: *P < 0.05. [file Image_2.TIF]
